# Modeling the Conversation with Digital Health Assistants in Adherence Apps: Some Considerations on the Similarities and Differences with Familiar Medical Encounters

**DOI:** 10.3390/ijerph20126182

**Published:** 2023-06-19

**Authors:** Anna Spagnolli, Giulia Cenzato, Luciano Gamberini

**Affiliations:** 1 Department of General Psychology, University of Padua, 35131 Padua, Italyluciano.gamberini@unipd.it (L.G.); 2 Human Inspired Technologies Research Centre, University of Padua, 35131 Padua, Italy

**Keywords:** digital health assistants, adherence applications, medical encounters, conversational agents, chatbot

## Abstract

Digital health assistants (DHAs) are conversational agents incorporated into health systems’ interfaces, exploiting an intuitive interaction format appreciated by the users. At the same time, however, their conversational format can evoke interactional practices typical of health encounters with human doctors that might misguide the users. Awareness of the similarities and differences between novel mediated encounters and more familiar ones helps designers avoid unintended expectations and leverage suitable ones. Focusing on adherence apps, we analytically discuss the structure of DHA-patient encounters against the literature on physician-patient encounters and the specific affordances of DHAs. We synthesize our discussion into a design checklist and add some considerations about DHA with unconstrained natural language interfaces.

## 1. Conversational Agents in Adherence Apps: Digital Health Assistants

Medication adherence is the process by which patients take their medications as prescribed. The World Health Organization has defined lack of adherence as a global problem of striking magnitude in terms of the number of patients affected and the costs to the health system [1]. Non-adherence includes late or non-initiation of the prescribed treatment, sub-optimal implementation of the dosing regimen, or early treatment discontinuation [2]. The factors leading to non-adherence include forgetfulness [3], miscommunication with the care provider, the complexity of medication, the patient’s wrong beliefs, and a lack of motivation [4]. Closely assisting patients who keep track of which medication to take and when to take it is then an essential condition for adherence.

Physicians cannot possibly provide immediate assistance to each patient under treatment, not even during medical trials. Instead, digital and mobile health applications (mHealth) can offer a scalable solution. The main goal of *adherence apps* is to ensure that patients take their medicines according to their treatment protocol. Thus, they send treatment reminders to the patients’ phones, informing them that the time has come to take a given medication. An associated goal is to collect reports of the patient’s symptoms since side effects are one of the causes of non-adherence [5]. They send prompts to update the patient’s diary, overcoming memory biases or gaps plaguing other data collection methods [6]. In 2015, more than 160 mobile telephone applications used one-way text messaging to improve medication adherence [7]. In 2019, Tabi et al. found 328 medication management apps in the iOS Apple App Store and the Android Google Play Store [8]. The World Health Organization encouraged using mHealth to achieve universal healthcare access, overcoming persistent infrastructural and health service delivery challenges [9]. Examples include distance education platforms [10], voice assistants for patients with poor dexterity [11], and chatbots to monitor medication overuse [12]. Mobile health applications can perform the monitoring tasks defined in advance by the physician and benefit from the accessibility, timeliness, and ubiquity of the mobile phone infrastructure [13]. Although they are no substitute for human healthcare experts and need quality safeguards [14], the alternative would be to leave thousands of patients without adequate support, dropping out of treatments for forgetfulness and isolation.

The transaction with the patient can be facilitated by using a conversational agent (CA). CAs are computer programs designed to simulate a conversation with humans; the patients interact with them either by choosing from a limited number of predefined input options or, in unconstrained natural language interfaces, with free text or speech [15]. When applied to health systems’ interfaces, they are called Digital Health Assistants (DHA). DHAs increase the effectiveness of health applications [16] for several reasons. First, they engage the patient more actively by requiring an answer; a meta-analysis by Wald et al. [7] found a 23% improvement in adherence if the patient was required to reply to the reminder, compared to a 4% improvement with one-way reminders. Second, DHAs adopt a friendly, empathic interaction modality, indulging the users’ need for social exchanges and entertainment to the point of being conceived as buddies [17]. Patients evaluate conversational agents positively and appreciate their effectiveness, accuracy, and accessibility [15]. 

Bickmore et al. [18] consider patient-facing conversational agents in healthcare and offer a wide-encompassing set of design principles that prevent inaccuracies and facilitate engagement. Recently, Richards, Vythilingam, and Formosa emphasized the ethical risk connected to artificial agents exhibiting or responding to human emotions [19]. One such risk is misrepresenting the DHA’s expertise [18]. The same concern is expressed by the EU Ethical Guidelines for Trustworthy AI, which demand that users be aware that they are interacting with an AI system [20]. 

The design of DHAs can take advantage of increasing user-centered research checking the features that improve user experience [21,22]. Laranjo et al. [23] reviewed 17 studies that assessed the user experience and efficacy of conversational agents with unconstrained natural language. Chew [24] examined 23 studies on artificial intelligence chatbots for weight loss, primarily focusing on strategies to personalize conversations and keep users engaged. Shan et al. [25] reviewed 11 studies on conversational agents for health communication, focusing on the chatbot’s ability to respond to the user with appropriate wording. 

Although these studies focused on the linguistic style or cultural background involved in the linguistic choices of the chatbot, we add to the work on user-centered conversational agents by focusing on the structure of the DHA encounters, particularly on the similarities with more familiar ones. From an ethnomethodological perspective, the structure of an encounter consists of the practices that implement its overall goal and allow its progression from phase to phase [26]. Like each new medium, which re-mediates the practices typical of previous media [27] mixing old and new action opportunities (“affordances” [28]), the DHA can evoke some encounters with which the patient is already familiar. Awareness of the similarities and differences between novel mediated and more familiar encounters might help designers avoid unintended expectations and leverage suitable ones. This paper is then an essay that analytically discusses DHA encounters in terms of their similarities with the structure of medical encounters and then synthesizes a set of guidelines to avoid misleading patients. Literature-based recommendations like these are common in human-computer interaction, where existing studies and literature are examined to inform a new product or interface. 

The paper is structured as follows: first, we will synthesize the knowledge on doctor-patient encounters in primary care, particularly follow-up visits, which bear affinities with DHA-patient encounters. Then, we will compare them to DHA encounters. Afterward, we will present our design recommendations, peppered with examples from a commercial application called Alira Health MyReco^®^, formerly PatchAi. For a larger project in 2021, we were granted access to all the conversation scripts related to headache treatment. The paper is not an assessment of that application, nor do the recommendations derive from it; we only use some dialogues from that application to illustrate more vividly the points we are progressively making. 

We hope this contribution will provide the preliminary context against which to analyze or design any specific DHA-patient conversation in adherence apps.

## 2. Similarities and Differences in the Structure of Physician- and DHA-Patient Encounters

The encounter with a DHA belongs to the domain of institutional encounters since it aims to achieve a specific goal and not to entertain the user [15]. More specifically, it belongs to the broader category of medical consultations; it gravitates around the patients’ conditions and includes a patient and a healthcare provider. The primary care encounter is the most generic type of medical consultation, on top of which other medical consultations build more specific goals and activities [29,30]. More specifically, the closest medical encounter to DHA-patient interaction is the follow-up visit [25,31] since the treatment has already been prescribed, and the patient updates its progress. 

**Figure 1:**
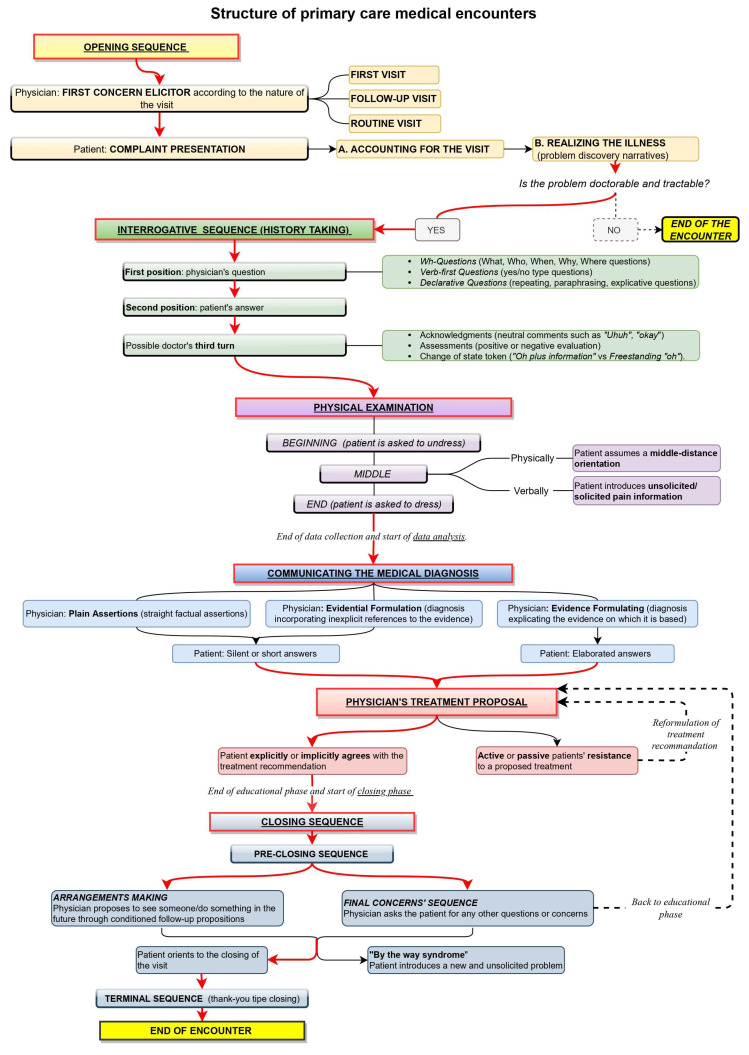
Diagram showing the sequential structure of primary care medical encounters in the ethnomethodological literature.

At a macro level, the sequential structure of a primary care encounter between the patient and physician has been observed to include six phases [29,32,33]: opening, history taking, physical examination, diagnosis communication, treatment proposal, and closing. A sketch of this sequential structure is offered in Figure 1. 

The first phase of the encounter is the opening [32,33], where the patient is invited to declare the reason for the visit with a prompt called ‘first concern elicitor’ [34]. In the case of a first visit, the description of the health trouble depicts it as worthy of attention from a health professional (“doctorable”) and amenable to treatment (“tractable”) [31]. If the doctor is thus convinced, the visit progresses to the next phase, the *history taking.* In the case of follow-up visits, instead, the physician asks directly about the state of the patient, whose health troubles have already been diagnosed and whose treatment has already been agreed upon. The first concern elicitor and how the patient reacts to it show whether the visit is a first or a follow-up. If the encounter represents an initial examination or the patient has an issue that has never been reported in previous encounters, the type of elicitor used to prompt the recount of the first concern is some variation of “What can I do for you today?” [29,35,36]. This open question implicitly demonstrates that the doctor does not know the interlocutor’s health status [30]. In follow-up visits, instead, the doctor prompts the recount of the complaint via resources such as “How are you?”, “How are you feeling?” or “How are you doing?” These prompts refer to the health state of the patient and ask for an update about it [30,31]. An inadequate prompt prompts relevant responses that the patient was unprepared to provide; consequently, the patient will try to realign the encounter’s footing. For instance, the doctor might elicit a follow-up, but the patient might reply, “This time, nothing to do with…” [37].

In the history-taking phase, the doctor collects information necessary to understand the patient’s problem/treatment efficacy by asking questions [38]. The patient might extend their answer to mildly propose a self-diagnosis, which the doctor decides to consider or ignore [39]. Deppermann and Spranz-Fogasy [40] observed that the question formats used are Wh-questions (“who,” “what,” “why,” and “when”), verb-first (e.g., “Did you …”) questions, and declarative questions. These formats vary in the level of the doctor’s presuppositions about the patient’s deeds. Declarative questions are more presupposing and are typical at the closure of a question-answer sequence; at that point, the doctor has already learned much about the patient’s state and wants to check their interpretation before moving on to the following argument [41]. Declarative questions can consist of verbatim repetitions of what the patient just said, thereby topicalizing the patient’s answer to demonstrate the relevance of the information provided and to invite the patient to further elaborate on it. Other declarative questions reformulate in more professional terms what the patient just said or extract information implicit in the patient’s answers. This phase might also include a physical examination [42], but it is irrelevant here. The doctor can acknowledge or assess the patient’s answer in a third turn [43]. Third-turn acknowledgments can be neutral (e.g., Okay), evaluative (e.g., Good), or change of state tokens showing wonder or surprise, such as “*Oh*”. The latter might imply ignorance, so they are used by the doctor when receiving information that is non-relevant to the medical business or of which the doctor cannot be aware [44].

After the history taking, the physician offers a *diagnosis*, backing it up with explicit or implicit reference to the evidence collected during the previous phases [45]. The patient can accept it with short answers or longer answers. The subsequent phase is the *treatment recommendation*. If the patient resists the proposed treatment, a negotiation starts where both parties, the patient and the doctor, express their arguments; the initial treatment might then be reformulated due to the patient’s preferences for specific types of treatment [46,47].

Once agreement is reached on the treatment, a *closing* phase follows when the visit reaches its end. This phase consists of a pre-closure process, including arrangements and final concern sequences [48,49,50,51,52], and a terminal exchange, including greetings and thanks. With the former, the next visit is arranged, dependent on health improvements; when the prognosis is positive, no time limit is set, but a “watchful waiting” strategy is applied [53]. Otherwise, a specific interval of time is mentioned to check the effectiveness of the therapy (test-of-time principle): once the established time interval has passed, if the symptoms worsen, the patient is assumed to return [54]. Arrangement-making also includes short-term agreements illustrating what will happen as the patient exits the room and is taken care of by another health operator, and it warrants “continuity of care”, i.e., that care does not end with the current visit. Final concern sequences, on the other hand, offer the patient the opportunity to share new problems, worries, or doubts left previously unexpressed in the conversation [55,56]. When this happens, a new issue is introduced, a phenomenon referred to in the literature as the “by the way syndrome.” [57] Although final-concern questions could greatly increase the patient’s satisfaction, the doctor’s way of asking final questions might discourage the actual introduction of additional concerns. For instance, indefinite pronouns such as “anything else” or “any” give the question a negative polarity and, as a result, suggest the expectation of a negative answer [55,56,58]. To elicit new concerns from the patient before concluding the visit, the doctor should phrase questions without negative response preferences, such as “Are there other things that you wanted to address today?” and orient themselves physically and visually toward the patient [48]. 

This framework is not deterministic but represents a reference against which every move is interpreted, including actions departing from such a framework [29,32,33]. Institutional encounters pursue a specific goal and are organized according to recurrent practices. Such practices have both a heuristic and an epistemic value: they provide ready solutions for recurrent issues and make the type of encounter recognizable to other members of the same culture [26]. Enacting the routines of a given encounter evokes the whole framework connected with that type of encounter, including its goal and the social role of the parties involved. It also makes particular courses of action and responses relevant. If these expectations do not fit the specific encounter, avoiding recourse to familiar practices or explicitly ruling out unsuitable implications is recommended. For instance, if the history taking is usually followed by diagnosis and treatment or low-level recommendations (e.g., resting), the patient may expect that reporting an acute symptom might induce the DHA to generate remedies. The DHA’s questions and third turns must then be formulated carefully to avoid giving the impression that they have some diagnostic relevance. 

### Similarities and Differences with DHA-Patient Encounters in Adherence Apps

Compared with the entire structure of a primary care medical encounter with a physician, the structure of the encounter with the DHA of adherence apps is less articulated. That type of DHA does not diagnose or make medical decisions; a physician defines the patient’s health problem (i.e., headache) and treatment before using the app. Therefore, the physical examination, the communication of the diagnosis, and the treatment proposal are not relevant phases in these DHA-patient encounters. The tasks of the DHA include generating timely reminders and data collection prompts, storing the health data collected, and being constantly present and available [59]. Therefore, the DHA-patient encounter in adherence apps shrinks compared to Figure 1 to only include three phases: the opening, the history-taking to monitor the patient’s health conditions and treatment compliance, and the closing. 

Moreover, the encounter does not always result from the patient’s initiative to visit the physician, as is commonly the case in physician-patient encounters. The DHA is programmed to contact the patient for prompts and reminders; in these cases, it is the DHA visiting the patient. The possibility that both parties start an encounter has implications for the management of the interaction since the starting party is cognizant of the reason for the encounter and should disclose it to facilitate mutual collaboration toward achieving the goal. Finally, the DHA can, at some point, simply work as an ordinary software assistant, ushering the patient to other parts of the application, such as the symptom history. This different role should also be considered while designing the structure of the encounter to provide a DHA that can assist the patient/user comprehensively yet unambiguously in the different functions of the application.

## 3. Design Recommendations Phase-by-Phase

The similarities and differences with physician-patient encounters highlighted above have important consequences for the dialogue structure of DHAs in adherence apps. Implementing conversational practices that the literature has already highlighted as familiar and recurrent in a specific kind of encounter would simplify the recognition of the encounter, its goals, and the roles of the parties involved. They would also suggest the projected trajectory of the interaction. At the same time, however, the differences highlighted in Section 2 suggest when familiar practices must not be implemented to avoid misunderstandings about the purposes and nature of the activity at hand. 

We, therefore, provide literature-based recommendations to design the sequential structure of a DHA-patient encounter in adherence apps. The recommendations derive from observational studies of physician-patient encounters and are critically adapted to the specificities of DHAs in adherence apps. By following those recommendations, designers can have a reference against which to create the sequential structure of their dialogue scripts instead of relying on their intuitive guesses.

**Figure 2:**
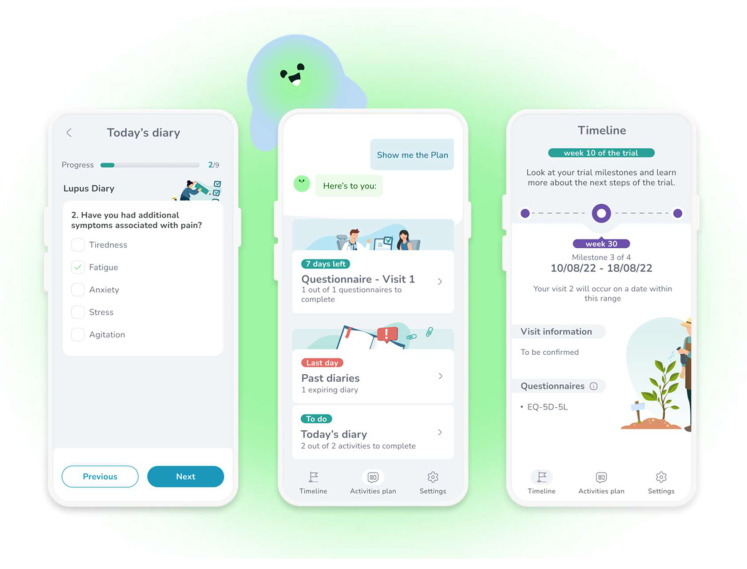
A few screenshots from the adherence application MyReco^®^, respectively a diary to enter the symptoms (**left**), a DHA-patient conversation offering access to several functions (**center**), and the achievements in the treatment/trial (**right**).

The recommendations will be illustrated with examples from a pre-review version of MyReco^®^ (Figure 2), an adherence app that simplifies a therapeutic regimen prescribed by a (human) doctor through reminders to take the therapy at the right time and in the prescribed dosage. As the patients complete their daily goals, they get recognition and rewards to boost their motivation and engagement. The application also actively involves the patients by having them provide health data (e-Diary) that the (human) physician will check at their convenience remotely or during period visits. Additional functionalities include planning check-ups (verifying that they took place) and accessing health education content. The examples will have a simpler layout than those displayed in Figure 2 because they derive from conversational scripts, not screenshots. Additionally, they are translated from Italian. 

We used examples from this application because we had access to its dialogue structure, and, like most adherence apps [13,15], it is text-based. As mentioned above, our goal is not to assess this app but to use its dialogue structures to illustrate our design recommendations. This app uses predefined answer options, i.e., the patient replies by choosing among the set of predefined answer options, for instance, a list of symptoms that need monitoring. Consideration will also be given to dialogues where input modalities allow free text.

### 3.1. Openings

**Figure 3:**
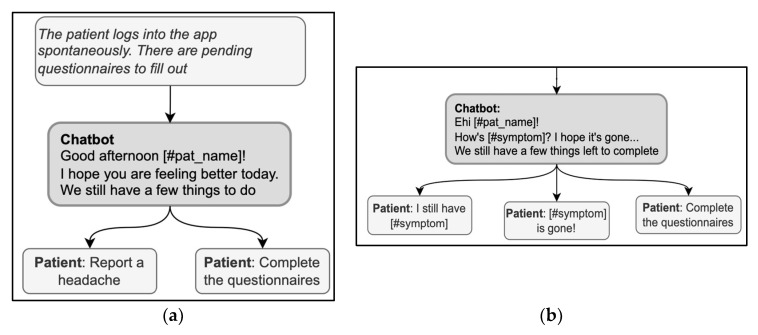
(**a**) (right) and (**b**) (left). Examples of sequences in the opening phase include first-concern elicitors.

The opening of a conversation should clarify the reason for the encounter. For instance, MyReco^®^ might start a conversation with the following message: “Hi (name of the patient)! Can you help me understand your overall wellness status?” In this message, the DHA greets and addresses the patient by name and immediately clarifies the reason for the encounter, i.e., checking the patient’s overall wellness status. When the patient starts the encounter, conversely, a first concern elicitor must be prompted, covering the possible reasons for the encounter. This way, the DHA could align with the patient’s goals and activate the correct conversational flow. The elicitor must be comprehensive to include all the possible reasons, either by listing them if there are a few or by using more general categorizations at first if there are many. Based on their knowledge of the user, the DHA can prioritize the more likely reasons and show them first. For instance, in the exchange in Figure 3a, the DHA produces a first concern elicitor, “we still have a few things to do,” that presumes the reason for the visit, i.e., completing some pending activity. This elicitor is then based on the knowledge of the patient’s schedule and history of previous encounters, so it is a good, personalized way to simplify the list of possible reasons for the encounter; at the same time, however, other possibilities are ruled out, e.g., checking the activity history, getting information, or opening the calendar. Instead, the response options must always cover all valid reasons for the encounter in a given treatment phase and do so stepwise to avoid overcrowding the screen with answer options.

Another remark emerging from the example in Figure 3a is a lack of consistency between some answer options and the elicitor. Specifically, the answer option “reporting a headache” is not an appropriate answer to a question asking for remaining pending activities. If reporting a headache is a rejection of the proposal of “having a few things to do,” then this should be explicitly stated in the answer option, for instance, “I cannot because I have a headache.” A similar lack of consistency can be observed in Figure 3b: the DHA uses two elicitors (“How is [symptom]” and “We still have a few things to complete”), and the answer options refer one to one elicitor and one to the other. For a message to avoid ambiguities, it should not combine more than one elicitor; in the example above, the DHA could have proposed to check the symptoms first and then complete any pending activities.

**Figure 4:**
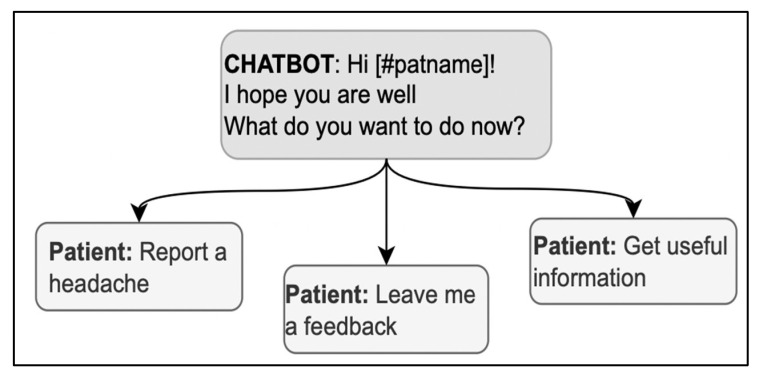
A sequence in the opening phase including a variation of “How are you?”.

Using the expression “How are you?” in the opening deserves particular caution; it is both a typical first concern elicitor in follow-up visits and a typical generic opening question used in any kind of social encounter, without any specific bearing on medical advice [60]; the patient might then be hesitant about whether or not the interlocutor is already collecting information or is just exchanging preliminary cordialities [61]. Thus, in case “how-are-you” is used as an elicitor, it should also include direct references to health issues to signal that serious information about health is being collected with that question (for example, “How is your headache, has it got any better?”) [37]. When used generically, the real elicitor can also be included in the message to avoid ambiguities; for example, in Figure 4, “How are you?” is immediately followed by a specific elicitor in the same message, “What do you want to do now?” Interestingly, the answer options contribute to the disambiguation by referring to the activities to do and retrospectively clarifying the question’s meaning.

The phrasing of the opening message contributes to the perception of the accuracy of the DHA. The opening phrase can imply shared knowledge of a pending activity; for example, “Are you ready to fill out the questionnaires?” A correct time reference demonstrates that the DHA recognizes the patients, accurately monitors their health, and keeps track of their activities. For example, the message “Good afternoon [#pat_name]! I hope you feel better today!” implies that the last encounter occurred the previous day and the patient was not well. It is necessary to ensure that the time reference is appropriate to the conversation in progress, especially if the patient accesses the application several times on the same day. In these cases, opening sentences with “I hope you feel better today!” can compromise the perceived accuracy of the monitoring. Proper openings for repeated encounters on the same day could be “I hope you are well” or “I am happy to hear from you.”

In conclusion, the opening sets the footing of the encounter, which in ethnomethodological tradition is the mutual establishment of the roles to take during a social interaction [62]. The proper footing in adherence apps represents the DHA as an assistant, tracking the patient’s health conditions and treatment compliance. Unambiguous and accurate references to the data collected and explicit mention of the reason for each encounter contribute to that representation. 

### 3.2. History-Taking

In a primary care visit, history taking is instrumental to enabling a diagnosis; in an adherence app, collecting information is a goal in itself and needs to be done as accurately as possible. Therefore, the phrasing of the questions is essential to accurately collecting information and adequately representing the DHA’s role. A question such as: “I’ll ask you some questions that will help me understand the problem” would be inconsistent with the latter because it seems to imply that the DHA can formulate a diagnosis. A more appropriate question would be, “I’ll ask you a few questions to keep track of this migraine episode,” making it clear that the purpose of the conversation is to collect data. 

The questions asked during history-taking presume some information about the patient’s lifestyle [63] and restrict the scope of the patient’s answers: they define their expected topic and suggested length [64]. They might also indicate the desirable polarity of the answer: optimistic questions, assuming positive expectations of the patient’s lifestyle or well-being, would make it more difficult for the patient to confess otherwise [16]. The same might also be true when the DHA is leading the history-taking phase since the interaction with conversational agents has proven to be subject to similar social norms (such as social desirability) as the interaction with humans [65,66]. Consequently, neutral phrasing is desirable when asking about the intensity of a symptom to avoid suggesting the answer to the patient.

**Figure 5:**
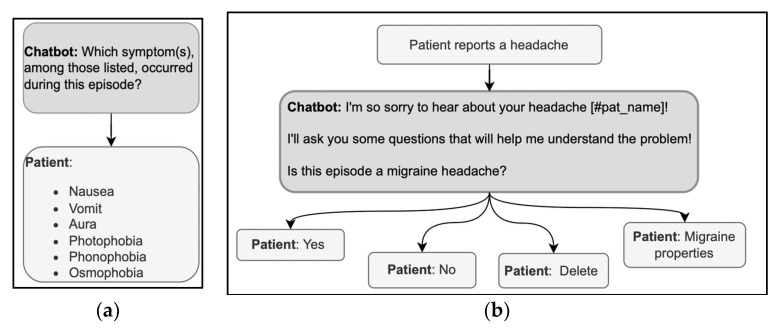
(**a**,**b**) Examples of sequences in the history-taking phase, using a Wh-question format (left) and a verb-first question format (right).

Questions in history-taking are usually Wh-questions such as What, When, Where, Who, why, or verb-first questions. In primary medical encounters, the Wh-questions are followed by freely formulated answers with which the patients can provide the information they possess and feel like sharing. The patient has no such freedom when answering a DHA with predefined answer options; thus, the answer options must be designed wisely. The relevant symptoms must be included, and an explanation of the criteria defining the range of relevant options should be provided. For example, in the exchanges illustrated in Figure 5a, the patient may have experienced symptoms other than those given in the list offered by the DHA. Thus, the DHA could clarify why there is only a limited set of options from which to choose or could provide non-specific answer options, such as “Other.” Furthermore, the symptoms could, unfortunately, be more than one; thus, the user could be allowed to select multiple response options instead of just one.

The other typical question format in history taking is verb-first, as in “Is this episode a migraine headache?” (Figure 5b). These questions show a higher level of presupposition on the doctor’s part than Wh-questions [40], but in medical encounters, the patient can expand the yes/no answer by adding clarifications [67]. With DHA using predefined response options, the patient can only choose between mutually exclusive choices (yes/no). When designing the conversation, it is then crucial that the user is allowed to correct their answer immediately, e.g., with the option “delete” in Figure 5b, to avoid having to start over. The correction could be implemented through commands external to the conversation or by asking for confirmation before analyzing a new topic. It is also helpful to start with a more general question and then delve into the details, a common practice through which doctors acknowledge or assess the patient’s reply [43]. Verbatim repetitions of the patient’s answer topicalize such an answer; they demonstrate the relevance of the information provided and invite further elaboration. A verbatim repetition could, for example, be inserted after the patient selects the ‘yes’ answer before asking for more details via further Wh-questions or verb-first questions. 

**Figure 6:**
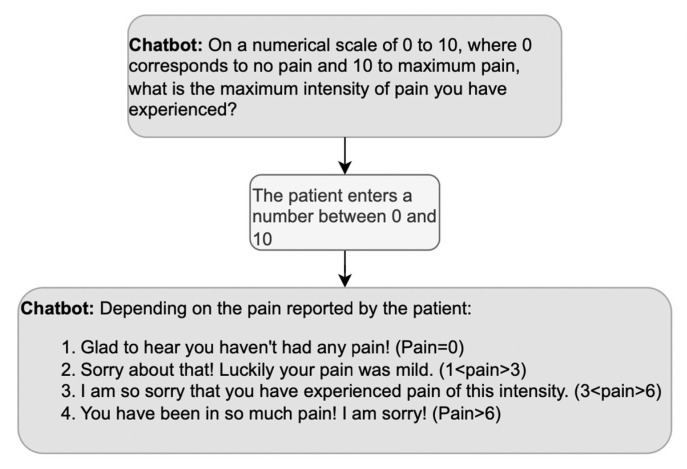
Example of a sequence including an empathic response to the patient’s answer; based on the answer, the DHA produces the corresponding empathic response.

What about positive or negative evaluations of the information provided by the patient during history taking? We mentioned above that doctors’ third turns in response to medical information are usually formulated neutrally since a display of surprise might imply ignorance. However, the DHA is not a doctor whose credibility relies upon not being surprised by the symptoms described; thus, the DHA’s evaluative turns can be appropriate to its role as a companion [59], as long as they show empathy. An example of empathic responses is reported in Figure 6, where the DHA selects among a set of four possible empathic reactions depending on the level of pain the patient declares. 

### 3.3. Pre-Closure and Closure 

To progressively reach the end of the doctor-patient encounter, the parties engage in pre-closure, mutually ensuring that the goal has been achieved: the plan of care is summarized, the patient’s understanding is checked, and a plan for interim contact is established [48,49,50,51,52,55]. The reason for the DHA-patient encounter is accurate data collection and compliance with the treatment schedule, so the pre-closure can acknowledge that the data has been obtained or that the reminder has been received. This acknowledgement can be done by thanking the patient for their collaboration, visualizing the data entered, and mentioning the subsequent encounter. Confirming the decisions made during the encounter allows any misunderstandings to be resolved before closure and signals that the encounter is heading toward closure. Indeed, the pre-closing phase warrants the patient an interactional space to share concerns left unexpressed that far in the interview [55,56], since the first concern elicitor in the opening phase solicited only one concern that became the focus of the conversation [14,58,68]. 

**Figure 7:**
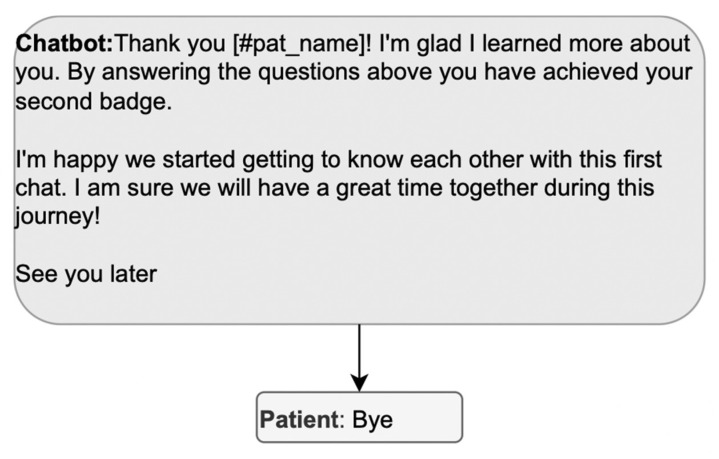
Example of a sequence where the patient cannot participate in the pre-closure.

The design must then ensure that the preclosing components are not missing. In Figure 7, the DHA unilaterally completes the pre-closure; the patient only participates in the closure (“bye”). To allow the patient to participate in the pre-closure, the DHA’s message could postpone the final greeting and propose some pre-closing activity, such as reviewing the data entered. Alternatively, the DHA could direct the patient to other activities with the app to spare the patient from exiting the conversation to open a new one right afterward. 

A careful design of the pre-closing should also be clear about the timing and conditions of the subsequent encounter. For example, phrases such as “See you later” issued in the evening seem to allude to the possibility that the reminder may be sent during the night. More specific phrases could be “I am available next time there is a report” or “Talk to you tomorrow to fill in the diary again.” Likewise, it might be helpful to remind the patient whether that exact activity will be resumed; in some cases, at the end of the onboarding flow, for example, it could be specified that that specific flow will not be repeated unless the patient wants to change the settings. 

**Figure 8:**
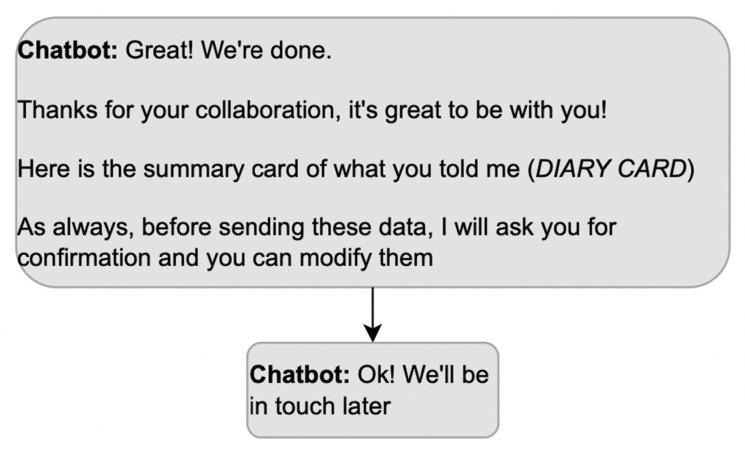
Example of a terminal sequence that does not allow the patient to participate.

Once arrangements are made and all possible additional reasons for using the app are exhausted, the closure becomes relevant; stopping the conversation without an explicit closure would make the status of the activity ambiguous. The presence of a terminal sequence allows the participants to close the meeting in progress, passing from a state of mutual involvement and reciprocal orientation to a state of inattention towards the actions of others, in which a participant’s turn does not make relevant a response. Without a terminal sequence, the patient may consider that some activities are still pending. Moreover, a terminal sequence must be built collaboratively by both interlocutors; in other words, it must be composed of a greeting from the DHA and a greeting from the patient. Instead, the exchange in Figure 8 misses the patient’s contribution to the terminal sequence, which is awkward in a conversation.

## 4. Discussion and Conclusions

In adherence apps, misunderstandings compromise the accuracy of the data collected, and managing them later forces the patients to endure a more extended interaction than is comfortable for their health conditions. Based on the conversational practices examined in the previous section, we can now organize a checklist to prevent misunderstandings between DHA and patients (Table 1). Managing misunderstandings involves: being explicit about the purpose and the state of the activity at each point during the encounter (Items 1, 1a, 1b, and 8); using unambiguous phrases (Item 3); keeping separate actions in separate messages with consistent answer options that do not confuse the patient as to what part of the question to answer (Items 3a and 3b); limiting the assumptions to what the DHA positively knows (Item 5); being transparent about the reason for omitting some answer options (Item 6); allowing prompt correction or confirmation (Item 5a); and avoiding to suggest positive answers (Item 7).

A parallel purpose of the checklist is to ensure that the encounter is set on the right *footing* regarding the mutual roles of the DHA and the patient and the connection of the current encounter with the previous ones. The task of the DHA is to be an assistant, collecting data to monitor the patient’s health condition for the physician. The DHA must then avoid self-attributing a clinical role. In the structure of the encounter, this footing is achieved in the DHA’s opening (Items 2, 2a, and 4) and in the third position turns (5b).

### 4.1. Unconstrained Natural Language Interfaces

If a DHA can accept the patients’ answers in the form of open text or speech, the intuitiveness of the inputting modality increases, and richer data can be collected, such as qualitative statements about the patient’s condition. In principle, these interfaces are especially appropriate for patients whose physical conditions might not allow them to focus on extended option lists to answer a question comfortably. Compared with DHAs accepting predefined message formats from the patient, however, they are more at risk of being misinterpreted. The reason is that closed answer options provide cues to retrospectively understand the meaning of the chatbot’s question and clarify which answers are pertinent. Instead, with unconstrained natural language interfaces, the mismatch between the interface capabilities and the users’ expectations needs to be deliberately managed and reduced (‘habitability’ [69]). Therefore, DHAs accepting natural speech should include targeted conversational practices to reveal and spot misunderstandings, called repairs (e.g., [70]). Repair practices point to the troublesome part of the previous speech/text in a way that signals what part of the message was unclear [71]. They can be used by the recipient of a message who feels they have not understood the speaker, or by the speaker who realizes they have been misunderstood. 

Additionally, DHAs need to collect accurate medical data. When collecting data via free text or free speech, DHAs could use third turns that rephrase the patient’s free answer to progressively find a clinically appropriate category as candidate understandings [71]. The chatbot should be transparent about the option range to avoid imposing one category. Indeed, this practice would be consistent with doctor-patient encounters, where declarative questions are used to reformulate with more professional terms what the patient just said or extract information implicit in the patient’s answers to test the doctor’s interpretation of the patient’s answer [41]. More recommendations on how to avoid being suggestive during medical encounters are wonderfully illustrated in [48].

### 4.2. Chats and Emergency Calls

As was mentioned above, DHAs in adherence apps do not use the patient’s inputted data to make diagnoses. Additionally, the data entered are not continuously monitored since physicians check them occasionally. The patients might not know what or who is behind the DHA. To prevent misrepresentations, applications such as MyReco^®^ make sure that the role of the DHA is clear from the onboarding phase by explicitly and simply explaining what the DHA can and cannot do and emphasizing that the app cannot be used to report an emergency hoping for immediate rescue. In addition to explicit disclaimers, the designers must assess how the DHA refers to itself, as mentioned in Section 3.2. Finally, the conversational model should avoid resembling emergency calls. In emergency calls, the opening sequence is minimized to enable fast assistance [72]; the first turn of the operator is a direct inquiry about the event that has led the caller to type the emergency number [73], followed by questions from the call-taker about the patient’s vital parameters [74]. So, a DHA starting directly with “How is your [symptom]?” followed by questions about the symptom’s acuteness might be similar to the structure of emergency calls. Constantly referring to what the DHA will do with the collected information can prevent misunderstandings; for example, a sentence like “How is your [symptom], so I can record it for you in your diary?” would clarify that the data are going to be used to update a diary and not be acted upon with immediate medical assistance. Additionally, preclosing sequences clarifying what was achieved in the conversation can prevent the expectation that the data entered will trigger immediate actions from some physicians. The designer might also consider including quick access to rescue services in the application, making it clear that they will address regular emergency dispatchers. If possible, advanced patient support can be considered, using risk prediction models for serious headache types to prompt clinical intervention if a specific response pattern is detected.

### 4.3. Limits 

Although the overarching structure and sequence of the medical encounter phases have been found to exist in different cultural settings, researchers in the fields of ethnomethodology and conversation analysis take cultural variations into account and conduct studies in diverse cultural settings to understand how cultural factors shape medical encounters. In this paper, we have not focused on these variants. For more information, a database of conversational studies on medical interactions can be found at https://emcawiki.net/Medical_EMCA_tag (accessed on 3 June 2023).

The examples we were able to provide derived from one adherence app; the reason is that we were granted access to the entire set of conversational flows prepared for the DHA in that app, unlike other apps that we could have just partially accessed as registered users. The examples from one adherence app do not diminish our contribution, which is not a case study and did not build on the specific structure of MyReco^®^, but to the more general structure of DHA encounters in their similarities and differences from doctor-patient encounters in primary care. However, other apps equipped to provide health recommendations or even diagnoses would include additional phases of a medical conversation.

Moreover, when designing DHA accepting free speech/text, additional guidelines should be derived from the analyses of the actual conversations with patients to identify issues in the mutual alignment. 

Finally, the recommendations here regard DHA in adherence apps, not the case in which the same app extends its goal to more proactive support like a virtual therapist. In that case, some of the empathic talks might be elaborated even more carefully to empower the patient [75,76].

### 4.4. Conclusions

In this work, we have outlined the possible similarities and differences between the overall structure of a DHA-patient encounter in adherence apps and familiar primacy care encounters, under the assumption that the latter belongs to the cultural context of the patients and can be evoked, along with a set of inferences and expectations, while interacting with DHAs. We have argued that awareness of which practices might trigger which expectations is a helpful step in designing DHAs, both in the interest of ethics and communication smoothness. Information about the DHA’s role and the meaning of its messages can also be achieved by adding instructions, help, and disclaimers. However, we believe that having adequate prompts and cues embedded in the conversational practices adopted by the DHA clarifies its role, and the expected tasks would be less wearisome to the end-user. Further work can address the cultural variations of the general framework outlined here and also test the improvement in terms of mutual understanding and transparency of the DHA’s role resulting from applying the guidelines presented here.

## Figures and Tables

**Table 1:** A checklist guiding designers to assess the consistency of the DHA-patient encounter’s structure.

Opening	
1	Does the caller explain the reason for the encounter at the opening (or re-opening after a pre-closure)? If the reason is not stated immediately, is it explicitly solicited by the called party within the opening phase? Alternatively, is the reason known to both parties (i.e., there is no other possible reason than one)?
1a	When the patient is the caller, does the DHA use a first concern elicitor, and is the answer to the first concern elicitor present and explicit?
1b	In DHAs using predefined answer options, do the answer options to the first concern elicitor cover all possible reasons for the visit?
2	Does the DHA address the patient in a way that acknowledges the recurrent nature of their encounters? For instance, by addressing them by their name, referring to their last encounter, or (if appropriate to the reason for the encounter) when the data was last collected
2a	Is the DHA’s first concern elicitor appropriate for the type of visit that is about to take place? (Note: If the activation of the DHA is a general one, without any clue about the reason for the visit, the first concern elicitor should be as vague as “What can I do for you?”; if some activities were postponed and need to be resumed as soon as the patient feels better, the first concern elicitor can be “How are you now?”)
3	Can the DHA’s initial greetings (how are you) be confused with a first concern elicitor, or does it specifically refer to the patient’s last reported condition or agreement?
3a	Does the DHA use one elicitor per message?
3b	Are the response options relevant (consistent) for the corresponding elicitor?
History taking	
4	Does the DHA begin an interrogation sequence only after completing the opening phase?
5	Does the DHA use Wh-questions to collect information it does not have and V1-Questions only for information already inputted and needing confirmation?
5a	Is the patient allowed to correct their inputted data at any point in the conversation, including right after inputting them?
5b	Does the DHA clarify what it does with the data and react empathically to the inputted data?
6	Does the DHA clarify the criterion, which makes some answers irrelevant (and then not present) among the answer options?
7	Does the DHA avoid formulating the question in a way that makes an optimistic answer desirable?
Closing	
8	Is there after history taking a pre-closing sequence (opened by DHA) that…
8a	… allows the patient to confirm what has been communicated to the DHA?
8b	…mentions or agrees about when (or if) the meeting will be repeated and for which activity?
8c	…offers the patient to carry out another activity instead of closing the encounter?
9	Is there a sequence that explicitly ends the encounter with thanks and greetings?

## Data Availability

No data was collected for this paper.
